# Dose-Dependent but Non-Interactive Effects of Ochratoxin A and Selenomethionine on Hepatic Lipid Metabolism and Oxidative Stress in Broiler Chickens

**DOI:** 10.3390/toxins17120568

**Published:** 2025-11-25

**Authors:** Szabina Kulcsár, Krisztián Balogh, Erika Zándoki, Edward Agyarko, Omeralfaroug Ali, Benjámin Kövesi, Ágnes Freiler-Nagy, András Szabó, Miklós Mézes

**Affiliations:** 1 Department of Feed Safety, Institute of Physiology and Nutrition, Hungarian University of Agriculture and Life Sciences, Szent István Campus, H-2100 Gödöllő, Hungary; 2 HUN-REN-MATE Mycotoxins in the Food Chain Research Group, Hungarian University of Agriculture and Life Sciences, H-7400 Kaposvár, Hungary; 3 Agribiotechnology and Precision Breeding for Food Security National Laboratory, Department of Physiology and Animal Health, Institute of Physiology and Nutrition, Hungarian University of Agriculture and Life Sciences, H-7400 Kaposvár, Hungary; 4 Department of Animal Hygiene, Herd Health and Mobile Clinic, University of Veterinary Medicine, H-1078 Budapest, Hungary

**Keywords:** ochratoxin A, selenium, broiler chicken, glutathione redox system, lipid peroxidation, fatty acid composition

## Abstract

This study examined the effects of ochratoxin A (OTA) exposure and graded dietary selenium (Se) supplementation on fatty acid (FA) composition and oxidative stress markers in the liver of broiler chickens. OTA is known to generate oxidative stress, promote lipid peroxidation, and affect the antioxidant system. Se, an essential trace element with antioxidant properties, may help counteract OTA-induced toxicity. In this short-term (5-day) in vivo feeding experiment, 21-day-old broiler chickens were divided into six groups, each with six birds: Control (diet free from Se), 0.3 mg/kg Se, 0.5 mg/kg Se, 2 mg/kg OTA, 2 mg/kg OTA + 0.3 mg/kg Se, 2 mg/kg OTA + 0.5 mg/kg Se. Our findings show that supplementing 0.3 mg/kg (*p* < 0.01) or 0.5 mg/kg Se (*p* < 0.001) in OTA-exposed birds significantly reduced the early oxidative stress markers (conjugated dienes and trienes) and significantly increased (0.3 mg/kg *p* < 0.01; 0.5 mg/kg *p* < 0.001) glutathione levels, indicating enhanced glutathione-dependent antioxidant protection. The treatments also significantly altered the ratio of monounsaturated and n6/n3 polyunsaturated FAs. OTA with 0.3 mg/kg Se supplementation significantly (*p* ˂ 0.05) reduced total unsaturation and FA average chain length. At a dose of 0.3 mg/kg, the interaction of Se and OTA altered the PUFA composition, while 0.5 mg/kg Se supplementation enhanced antioxidant defense and reduced lipid peroxidation. These results highlight the dual but separate role of Se, where inadequate doses may enhance OTA toxicity, while optimal supplementation may have a protective effect on hepatic lipid homeostasis. These findings can be used in the future progress of the mitigation strategy against OTA exposure in poultry nutrition.

## 1. Introduction

Due to global warming, climate change within European temperate regions makes them more suitable for the growth and spread of toxigenic molds, including *Aspergillus*, *Penicillium*, and *Fusarium*. Rising temperature and periodic droughts expand fungal habitats, increasing mycotoxin contamination in crops [1]. Such contamination poses risks to agriculture, as contaminated animal feed can lead to production losses or toxic effects in livestock depending on the dose [2]. Ochratoxin A (OTA), a secondary metabolite of certain *Aspergillus* and *Penicillium* species, is one of the most prevalent nephro- and hepatotoxic mycotoxins contaminating poultry feeds worldwide [3]. Due to carry-over effects, OTA residues can also be found in animal-derived foods such as pork, poultry, milk, and cheese, contributing to human exposure [4].

Broilers are markedly sensitive to OTA toxicity; exposure to 0.4–0.8 mg OTA/kg feed from day 1 to week 5 has been associated with significant reductions in body weight, feed intake, thymus weight, red and white blood cell counts, and immune response, along with increased mortality [5]. In the European Union, the recommended maximum level of OTA in cereal-based and poultry feeds is 0.10 mg/kg, as established by Commission Recommendation 2006/576/EC on the presence of OTA in products intended for animal feeding [6]. The toxic effect is the result of several mechanisms of action, such as the inhibition of protein synthesis, DNA and mitochondrial damage, and modulation of signaling pathways [7]. Besides these, OTA promotes the formation of reactive oxygen species, which can cause lipid peroxidation and oxidative damage to cellular components, including membrane lipids [8,9]. In liver (HepG2) cells, incubation with 100 μM OTA reduced cellular GSH levels, one of the most important intracellular antioxidants, and influenced ROS production in a dose-dependent manner, which may contribute to the hepatotoxic effects of OTA [10]. Alterations in the hepatic fatty acid (FA) composition and lipid peroxidation markers reflect these oxidative processes, disrupting membrane integrity, cellular permeability, and enzyme activity [11], primarily altering the metabolism of the polyunsaturated FAs of the glycerophospholipidome. Lipid peroxidation depends on the rate of unsaturation of FAs because the higher number of unsaturated double bonds in the fatty acyl chain increases its susceptibility to peroxidation. Peroxidized FAs in the cell membranes, particularly in mitochondrial membrane, may cause the release of oxygen free radicals and induce oxidative stress in the liver [12].

Consistent with these findings, studies have reported that low-molecular-weight antioxidants, such as tocopherol, ascorbic acid, and phytogenic bioactive feed additives can decrease lipid peroxidation and mitigate OTA-induced damage [13,14]. Selenium (Se) is an essential trace element that plays a key role in protecting against oxidative stress, both as a component of selenoproteins, particularly the glutathione peroxidase (GPx) isoenzymes, and by supporting non-enzymatic antioxidant mechanisms, such as vitamin E and reduced glutathione (GSH) [15]. Se has immunomodulatory effects similar to some bioactive plant extracts [16,17] and inhibits apoptosis [18]. Through these mechanisms, Se can reduce or even prevent the oxidative stress caused by OTA. Additionally, oxidative stress and Se modulate activation of liver progenitor cells through the VEGF-FGF signaling mechanism [19]

Se has an antagonistic effect against the toxicity of mycotoxins, such as aflatoxin A, T-2 toxin, deoxynivalenol, and OTA [20]. The protective effect of Se suggested provides its antioxidant properties through the Nrf2 signaling pathway. However, another mode of action of Se through the JAK1/STAT3 signaling has also been proposed and experimentally proven [21]. Selenomethionine could regulate the Nrf2/Keap1 and PI3K/AKT pathways to reduce the hepatotoxicity induced by OTA in chicken [22]. It means that selenomethionine activates signaling pathways that counteract OTA-induced oxidative stress. Moreover, nutritional factors such as Se supplementation can substantially modify the composition and metabolic capacity of the gut microbiota, which in turn may influence the severity and characteristics of OTA-induced liver injury [23]. There is no data in the literature about the effect of OTA on hepatocellular membrane FA composition, as well as in relation to selenomethionine. However, some studies propose potential FA alterations in meat and liver in OTA toxicity [24,25], but not about its interaction with Se supplementation.

Based on these considerations, the aim of this research was to evaluate the changes in lipid peroxidation, efficiency of the glutathione redox system, and total phospholipid FA composition resulting from OTA-induced oxidative stress. Another aim was to assess the effectiveness of the Se supplementation in mitigating potential adverse effects caused by OTA exposure such as oxidative stress and FA composition.

## 2. Results

### 2.1. Body Weight, Liver Weight

Neither Se complementation, OTA separately, nor OTA + Se combinations significantly affected BW after 5 days. Liver weights (absolute and relative) were also similar among the groups (Table 1).

### 2.2. Fatty Acid Profile of Total Hepatic Phosphatides

The fatty acid profile of total hepatic phosphatides is shown in Table 2.

As evaluated with ANOVA and Tukey’s post hoc test, within the saturated FAs, the lower level of Se supplementation (0.3 Se/kg feed) increased the C20:0 (arachidic acid) proportion above the control, while both levels of Se supplementation increased the C22:0 (behenic acid) proportion above the OTA mean. The Se-supplemented groups generally exhibited higher C22:0 levels, whereas the OTA-exposed groups showed a decrease in its content. Interestingly, total saturation level was lowest in the Se 0.5 group, while the highest value was shown in the OTA-Se 0.5 group.

Among the monounsaturated FAs, the proportion of C16:1 n-7 (palmitoleic acid) was higher in the Se 0.3 group as compared to the control, while the proportion of C18:1 n-9 (oleic acid) in the OTA-Se 0.3 group exceeded the control. An increase in total monounsaturated FAs was observed in both the Se 0.3 and OTA–Se 0.3 groups, merging the effects of both treatments when compared to the control.

In the n-6 FA family, the arachidonic acid (C20:4 n-6) proportion was lowered by Se 0.3 alone and in combination with OTA as well, as compared to the control, while the adrenic acid (C22:4 n-6) proportion was only lowered by the combination of Se 0.3 and OTA. Docosapentaenoic acid (C22:5 n-6) was decreased by the treatments Se 0.3, Se 0.5, and OTA-Se 0.3 below the control. Only the OTA-Se 0.3 group showed a substantial decrease in the sum of all n-6 FAs compared to the control (Table 3).

In the n-3 FA family, OTA-Se 0.3 lowered the docosapentaenoic acid (C22:5 n-3) proportion below the control, while for docosahexaenoic acid (C22:6 n-3), the Se 0.3, Se 0.5, and OTA-Se 0.3 treatments had the same effect. With respect to the total n-3 FAs, both the low-dose Se and OTA-Se 0.3 treatments lowered the proportion compared to the control.

The unsaturation index (UI) was lowered by OTA-Se 0.3, as compared to the control. A fully identical pattern was found for the average FA chain length (ACL).

Analyzing the Se and OTA effects as factors with the GLM model, the cases where Se did not provide a significant effect were C20:0 and the sum of saturated FAs, while the effect of OTA was more diverse on the FA profile (Table 3); it significantly affected C22:0, C20:4 n-6, total n-6, PUFA, and UI. Significant interactions among Se and OTA were not found on the lipid profile, indicating the agents’ separate effects.

### 2.3. Antioxidant and Lipid Peroxidation Parameters

**Figure 1:**
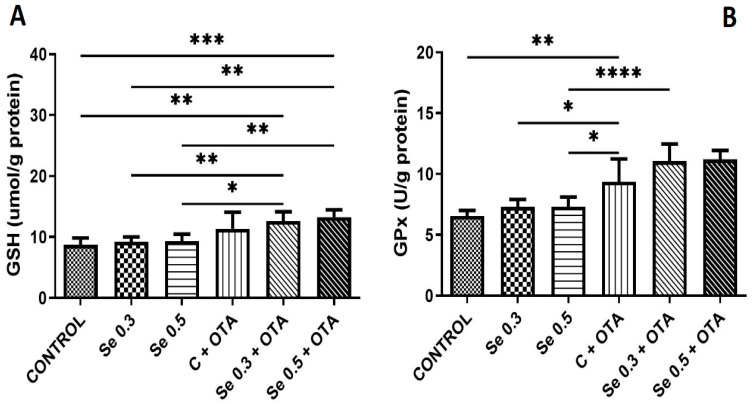
Development of markers of glutathione-based antioxidant protection by day 5 of the experiment (means of 6 individual data ± standard deviation). (**A**) Reduced glutathione; (**B**) glutathione peroxidase (means of 6 individual data ± standard deviation). Control: no OTA or Se; Se 0.3: 0.3 mg/kg Se; Se 0.5: 0.5 mg/kg Se; OTA: 2 mg/kg OTA OTA-Se 0.3: 0.3 mg/kg Se and 2 mg/kg OTA; OTA-Se 0.5: 0.5 mg/kg Se and 2 mg/kg OTA. * *p * <  0.05; ** *p * <  0.01; *** *p * <  0.001; **** *p * <  0.0001.

The GSH concentration was elevated by OTA exposure alone and in combination with both low and high Se complementation above the control. However, Se supplementation did not change the GSH content. Se supplementation at both levels did not change the GPx activity. OTA exposure alone and in combination with Se revealed higher GPx activity than the control, and this difference was also significant as compared to Se-supplemented groups (Figure 1).

**Figure 2:**
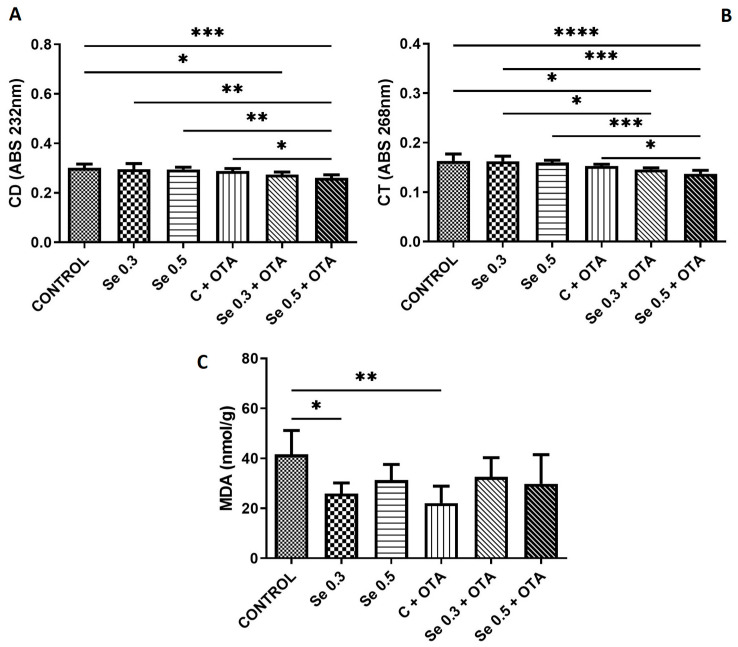
Changes in the markers of lipid peroxidation by day 5 of the experiment (means of 6 individual data ± standard deviation). (**A**) Conjugated dienes; (**B**) conjugated trienes; (**C**) malondialdehyde. Control: no OTA or Se; Se 0.3: 0.3 mg/kg Se; Se 0.5: 0.5 mg/kg Se; OTA: 2 mg/kg OTA; OTA-Se 0.3: 0.3 mg/kg Se and 2 mg/kg OTA; OTA-Se 0.5: 0.5 mg/kg Se and 2 mg/kg OTA (* *p*  <  0.05; ** *p*  <  0.01; *** *p*  <  0.001; **** *p*  <  0.0001).

While 0.5 mg/kg Se supplementation and OTA exposure alone did not alter CD and CT levels, their combined application resulted in significant reduction compared to the control, Se 0.3, Se 0.5, and OTA alone groups. A reduction in CD and CT levels was also observed in the group receiving 0.3 mg/kg Se + OTA compared to the control and, in the case of CT, even compared to the Se 0.3 group. Both 0.3 mg/kg Se supplementation and OTA exposure alone reduced MDA concentrations relative to the control (Figure 2).

Similarly to the effects’ analysis, GLM was invented to analyze possible interactions of Se and OTA. Effects are summarized in Table 4.

The results indicate the separate effects of Se and OTA, the latter significantly affecting all parameters, while Se had no effect on GSH and MDA. Remarkably, we did not find any interaction between the two agents.

### 2.4. Group Classification (sPLS-DA)

**Figure 3:**
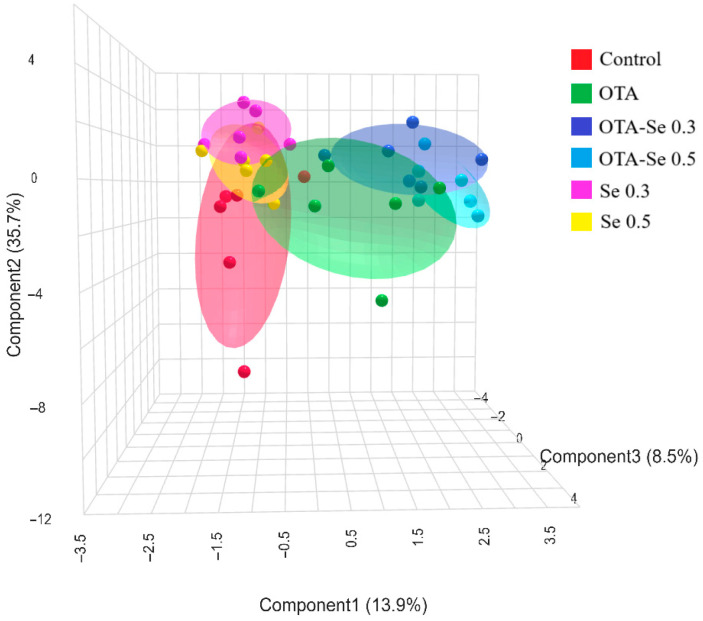
3D sPLS-DA score plot of the experimental groups (data: phospholipid fatty acids, lipid peroxidation, and antioxidant markers). Control: no OTA or Se; Se 0.3: 0.3 mg/kg Se; Se 0.5: 0.5 mg/kg Se; OTA: 2 mg/kg OTA; OTA-Se 0.3: 0.3 mg/kg Se and 2 mg/kg OTA; OTA-Se 0.5: 0.5 mg/kg Se and 2 mg/kg OTA.

Performing sPLS-DA analysis revealed efficient spatial separation of the six experimental groups (Figure 3). In the figure, the OTA and Se groups’ effective spatial separation is clearly visible.

**Figure 4:**
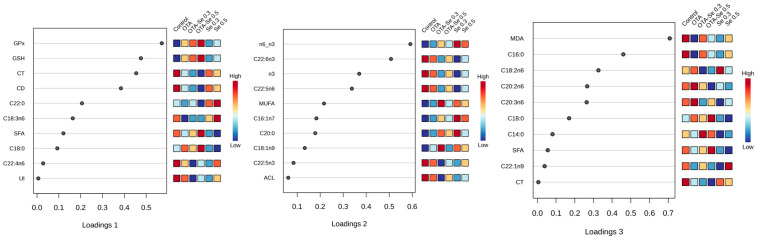
Loading plots of the 3D sPLS-DA score plot, highlighting the biological components’ efficacy in group formation. Colors indicate variable abundance, with dark brown representing high values and blue representing low values.

In the frame of the sPLS-DA analysis, we checked the top 10 most powerful loading items contributing to the spatial separations’ components. Figure 4 provides these loading plots with the most powerful biological variables. For component 1 the variables of the highest contribution to the separation were mostly antioxidants (GPx, GSH) and early lipid peroxidation markers (CD, CT); those for component 2 mostly contained FA groups (e.g., n3, MUFA), while those in component 3 were individual FAs and MDA.

## 3. Discussion

The 5-day exposure to 2 ppm OTA, a relatively high dose, was chosen to capture early oxidative stress responses and initial changes in lipid metabolism without causing severe systemic toxicity or secondary organ damage, allowing assessment of the primary interactions between OTA and Se. The current study indicates that dietary Se supplementation at both 0.3 and 0.5 mg/kg dosages modulates oxidative stress biomarkers and modifies the FA composition of hepatic membrane phospholipids in broiler chickens exposed to OTA. While the interaction between OTA and Se is known, its impact on membrane phospholipids remains unexplored. OTA modifies the hepatic xenobiotic biodetoxification mechanism and it may have an effect on OTA and Se interaction due to activation or suppression of different signaling pathways [26]. In addition, growing evidence suggests that the gut microbiota, through its close metabolic association with the liver, can influence hepatic phospholipid composition; thus, microbiota-mediated modulation of lipid metabolism may further interact with OTA and Se exposure [27,28]. Besides Se, other dietary strategies were proposed against OTA toxicity, such as vitamins and medicinal herb extract [29]. However, Se is a natural component of the regular diet of chicks, and its supplementation can be easily integrated into feeding practices.

### 3.1. Alterations in Hepatic Membrane Lipid Profile

The physiological dose of Se (0.3 mg/kg feed) addition modified the saturated and monounsaturated FA composition by increasing levels of arachidic acid (C20:0) and palmitoleic acid (C16:1 n-7). OTA exposure selectively reduced behenic acid (C22:0) levels. This decrease has also been reported in the liver of rats following zearalenone exposure [30] and in rabbits treated with fumonisin B1 [31], the latter being attributed to the inhibition of ceramide synthesis, as behenic acid is an essential component of sphingomyelins. In the case of OTA, such an effect has not been described yet; however, by influencing the transcription of several proteins [32], it may disrupt ceramide or sphingomyelin metabolism, which could indirectly affect behenic acid levels. While 0.5 mg/kg Se alone reduced total SFA content, combining this dose with OTA resulted in an increase, suggesting that OTA counteracts the Se-induced reduction of specific SFAs, possibly by modulating elongase and desaturase activity, as observed in Se-treated [33] and GPx1-overexpressing mice [34]. This suggests a complex, potentially antagonistic interaction (*p* = 0.007) between OTA and Se in the regulation of hepatic lipid metabolism and membrane integrity [35].

While OTA exposure alone did not significantly affect PUFA levels, the combined treatment with 0.3 mg/kg Se led to decreases in both n-6 and n-3 FAs. This result suggests that OTA exposure influenced PUFA profiles in the liver membrane, but only during 0.3 mg/kg Se supplementation, potentially by affecting susceptibility to lipid oxidation [15]. These results indicate that the simultaneous presence of OTA and Se exerted marked effects on lipid metabolism, leading to enhanced PUFA degradation, as reflected by the decreased unsaturation index (UI) and average chain length (ACL) observed in the OTA–Se (0.3 mg/kg) group, in line with the recognized vulnerability of long-chain PUFAs to oxidative damage [36].

### 3.2. Changes in Oxidative Stress Markers

A 5-day exposure to 2 mg/kg OTA increased GPx activity compared to the control group, which is consistent with a previous study reporting significantly higher GPx activity in broilers treated with 1 mg/kg OTA [37]. Additionally, elevated GPx activity was observed in the groups treated with 2 mg/kg OTA + 0.3 mg/kg Se, while increased GSH concentrations were found in both the 2 mg/kg OTA + 0.3 and 0.5 mg/kg Se groups. These findings suggest that Se supplementation in the presence of OTA enhances the glutathione redox response, likely by increasing Se-dependent GPx activity [38], as part of a specific adaptive response to OTA-induced oxidative stress. Moreover, OTA-induced oxidative and ER stress pathways may also be modulated by certain gut-derived microbial and cellular processes, which can exert effects similar to those of Se observed in the current study [39]. In the case of another hepatotoxic mycotoxin, supplementation with 0.4 mg/kg Se similarly mitigated AFB1-induced oxidative stress in the broiler liver by enhancing GPx activity and elevating GSH content [40].

The levels of early lipid peroxidation markers (CD and CT) did not change after feeding 0.2 mg/kg OTA compared to the control group. Similarly, in a previous study, these parameters showed no significant changes in the liver after 21 days of exposure to a 1126 μg OTA/kg feed [41]. However, supplementation with 0.5 mg/kg Se in addition to OTA resulted in a significant decrease in CD and CT levels compared to the OTA-only group. This effect is likely attributable to the antioxidant properties of Se, as further supported by the elevated GSH concentrations observed in this treatment group compared to the control. The level of the terminal phase marker of lipid peroxidation (MDA) decreased in the low Se (0.3 mg/kg) and OTA-treated group. The effect of Se on MDA formation is due to the well-known antioxidant effect of Se [42]. This effect was similar in a study with chicken breast muscle when MDA was reduced with 0.4 mg/kg Se, compared to controls [43]. In the OTA-treated group, this decrease was a consequence of reduced unsaturation of FAs (mainly C22:0). The altered FA composition may influence the intensity of lipid peroxidation processes due to different susceptibility to oxidation [44].

Based on the sPLS-DA analysis, Se and OTA treatments in broilers primarily affect antioxidant defenses and early lipid peroxidation markers, followed by changes in FA composition and lipid peroxidation end products. These findings reflect a complex interaction between oxidative stress and lipid metabolism, further highlighting their biological significance as key differentiating variables among treatment groups. Metabolomic analysis of the effect of OTA and its interaction with aflatoxin M1 was investigated previously, but not with Se [45].

Overall, 5 days of 2 mg/kg OTA exposure alone did not markedly alter the FA profile or antioxidant responses in broilers. However, in combination with the generally used 0.3 mg/kg Se content of feeds, OTA induced significant changes in lipid metabolism, as reflected in a decrease in both n-6 and n-3 FAs, as well as in a decrease in the unsaturation index and average chain length. These alterations were accompanied by increased GSH content and GPx activity, suggesting that the simultaneous presence of OTA and Se triggers parallel effects: while enhancing antioxidant responses, it also promotes lipid profile disturbances, compromising liver membrane integrity and redox balance. Based on the GLM analysis, the effects of OTA and selenium appeared independent, which may be due to the activation of different pathways. In another short-term experiment [46], OTA stimulated the expression of xenobiotic detoxification enzymes (e.g., CYP1A2), whose metabolic by-products can promote oxidative membrane damage. In contrast, Se acted through selenoprotein-dependent antioxidant mechanisms, activating the glutathione redox system (*GPX4*, *GSS*, *GSR*), enhancing cellular redox homeostasis, rather than modulating OTA detoxification pathways (AHR–CYP1A2). These different mechanisms may explain the lack of a strong interaction and the limited protective efficacy of Se at physiological levels against OTA-induced hepatic alterations. Multivariate analysis further highlighted antioxidant capacity and lipid peroxidation markers as key discriminating factors between treatments, underlining the central role of redox balance in the effects of OTA and Se. The results differ from previous studies [20], which may be due to short-term exposure and Se supplementation at the regulatory limit (0.3 and 0.5 mg/kg) concentrations, but highlight a possible negative dose-dependent effect of Se supplementation in OTA-contaminated feed.

## 4. Conclusions

The present study demonstrates that ochratoxin A (OTA) and selenium (Se) exert complex and dose-associated effects, but no proven interactions in broiler chickens, particularly in relation to lipid metabolism and oxidative stress responses. Separate OTA and Se effects’ parallel onset refers to divergent modes of action of these agents in the liver. An amount of 0.3 mg/kg Se was found to be ineffective against the effects of 0.2 mg/kg OTA in terms of hepatic PUFA levels, while 0.5 mg/kg Se supplementation effectively mitigated the effects of OTA by enhancing antioxidant defense, thus supporting membrane lipid integrity.

## 5. Materials and Methods

### 5.1. The Experimental Conditions

A total of 36 newly hatched Cobb 500 broiler cockerels were obtained from a hatchery (Babádi Hatchery Ltd., Ócsa-Felsőbabád, Hungary) and divided into six groups (*n* = 6, in 2 replicates). The birds in each group were housed in pens bedded with pine wood shavings. The short-term OTA exposure trial lasted for 5 days and was conducted on 21-day-old birds. The short-term exposure was selected based on our previous protocol. The short period allowed us to follow the early changes in OTA exposure and Se supplementation, and ageing did not modify the effects. Experimental groups were formed at 20 days of age, during which the birds were individually weighed to ensure that the average body weight within each group did not differ by more than 5%. Feed and drinking water were provided ad libitum under a natural light regimen (15 h light–9 h dark). The nutritional composition of the diet was as follows: 88.76% dry matter, 21.25% crude protein, 4.25% crude fiber, 1.10% calcium, 1.12% lysine, 0.38% methionine, 0.89% methionine + cysteine, 0.48% available phosphorus, 0.25% sodium, and 12.66 MJ/kg metabolizable energy (Table S1). The feed excluded mycotoxin binders and coccidiostats. The following groups were established: control (diet free from OTA and Se), Se groups (received diets supplemented with organic Se at either 0.3 mg/kg or 0.5 mg/kg concentrations), and OTA groups (fed diets contaminated with 2 mg OTA/kg, as well as the same OTA level with either 0.3 or 0.5 mg Se/kg. Se levels in broiler chicken feed differ across countries, typically ranging from 0.1 to 0.5 mg/kg, reflecting regional regulatory differences and evolving nutritional recommendations. Among these different concentrations, the 0.3 mg/kg dietary Se level was selected based on earlier studies that showed optimal performance at this concentration [47,48]. Moreover, the maximum permitted Se concentration in feed according to EU regulations is 0.5 mg/kg [49], so this higher concentration was also used in this experiment to enhance antioxidant defense mechanisms. Liver samples were collected from six birds per treatment group within 120 h of the start of OTA exposure and the samples were then stored frozen at −70 °C for at least one month until further analysis.

### 5.2. Ochratoxin A Contamination and Selenium Supplementation

OTA was produced by inoculating sterile ground corn substrate with *Aspergillus albertensis* strain SZMC 22107, obtained from the Microbiological Collection of the University of Szeged, Hungary. The fungal strain was cultured on potato dextrose agar (PDA) plates at 25 °C for eight days. Subsequently, the surface of the PDA was rinsed with deionized (DI) water to prepare the inoculum suspension. For substrate infection, 1 kg of corn matrix was moistened with 400 mL of DI water, sterilized, and cooled. Then, 2 mL of the fungal inoculum, adjusted to an optical density (OD) of 2.0, was thoroughly mixed into the corn substrate. The inoculated substrate was incubated at 25 °C for 30 days. Following incubation, OTA concentration in the substrate was quantified in triplicate by high-performance liquid chromatography (HPLC) with fluorescence detection, following immunoaffinity cleanup using OchraStar^®^ IAC columns (RomerLabs, Tulln, Austria), as described by Stroka et al. [50]. The calculated amount of OTA-contaminated substrate was mixed into the experimental feed based on its measured OTA content, and the final OTA content of the experimental feeds was also determined in triplicate (Table 5).

An organic Se (selenomethionine) preparation, Selisseo^®^ (Adisseo, Antony, France), containing 100% hydroxy-selenomethionine (OH-SeMet), was used. The Se concentration of the feed sample was determined in triplicate by inductively coupled plasma mass spectrometry (ICP-MS) (Perkin Elmer NEXION 2000, Perkin Elmer, Waltham, MA, USA). For sample preparation, 0.5 g of homogenized sample was weighed into Teflon digestion vessels (CEM MARS XPreSS, CEM Corp., Matthews, NC, USA). Each sample was treated with 5 mL of nitric acid (HNO_3_) and 5 mL of hydrogen peroxide (H_2_O_2_). The vessels were sealed and subjected to microwave digestion according to the following program: 35 min heating time to a final temperature of 200 °C, followed by 50 min holding time at a maximum power of 1700 W. After digestion, the samples were cooled and diluted with ultrapure water to a final volume of 25 mL. A fivefold dilution was performed before analysis. Blank and quality control samples underwent identical preparation. Teflon digestion vessels were cleaned with a 0.15 M HCl solution between runs to ensure analytical accuracy. The amount of Se preparation was mixed into the experimental feed, and the final Se content of the experimental feeds was determined in triplicate (Table 5).

### 5.3. The Analysis of Oxidative Stress Markers

Conjugated dienes (CD) and trienes (CT) were determined in triplicate by absorption spectra after extraction of liver lipid content in trimethylpentane [51]. Malondialdehyde (MDA) concentrations were determined in triplicate by complexation with 2-thiobarbituric acid in an acidic medium at high temperature [52]. GSH levels were measured in triplicate by the method of Rahman et al. [53], and GPx activity was measured in triplicate by the method of Lawrence and Burk [54]. GSH content and GPx activity were normalized to the protein concentration of the supernatant fraction, which was determined using the Folin–Ciocalteu phenol reagent method [55].

### 5.4. Fatty Acid Profiling of Tissue Phospholipids

Liver samples were homogenized in a chloroform–methanol (2:1, *v*/*v*) solution at a 20-fold volume, followed by lipid extraction according to the method of Folch et al. [56]. High-purity solvents (Merck-Sigma-Aldrich, Schnelldorf, Germany) were used, with 0.01% *w*/*v* butylated hydroxytoluene added to prevent FA oxidation. Following Leray et al. [57], 10 mg of total extracted lipids was applied onto glass chromatographic columns packed with 300 mg silica gel (230–400 mesh) for lipid fractionation. Neutral lipids were eluted using 10 mL chloroform, followed by 15 mL acetone–methanol (9:1, *v*/*v*), whereas total phospholipids (PLs) were eluted with 10 mL pure methanol. The collected PL fraction was evaporated under a nitrogen stream and subsequently methylated via the base-catalyzed sodium methoxide (NaOCH_3_) method described by Christie [58]. The determination of FA methyl esters (FAMEs) was performed as previously described [59]. Results are expressed as weight percentages of total FAMEs.

### 5.5. Statistical Evaluation

Intergroup differences were tested with analysis of variance and Tukey’s post hoc test. The selective effects of Se and OTA on biological variables were analysed with GLM, and OTA and Se were enrolled as fixed factors into the models. IBM SPSS 29 (2024) and GraphPad Prism 9.5.2 (GraphPad Software, San Diego, CA, USA) were used for the analysis. Furthermore, by involving FA, lipid peroxidation, and antioxidant data in a joint approach, sparse Partial Least Squares Discriminant Analysis (sPLS-DA) was performed with the Metaboanalyst GUI (version 2) [60].

### 5.6. Ethical Issues

During the experiment, the guidelines set by the European Communities Council Directive (86/609 EEC) were followed. The experimental protocol was approved by the Food Chain Safety, Land Use, Plant and Soil Protection and Forestry Directorate of the Pest County Governmental Office (PE/EA/1964-7/2017) with the lowest number of animals possible for an accurate statistical analysis.

## Figures and Tables

**Table 1:** Body and liver weights of broiler chickens present as mean standard deviation (*n* = 6).

Group	Control	Se 0.3	Se 0.5	OTA	OTA-Se 0.3	OTA-Se 0.5
body weight (g)	1023.33 ± 135.23	1046.67 ± 137.28	1086.67 ± 87.79	1053.33 ± 145.52	1090.83 ± 73.17	1079.17 ± 62.56
liver (g)	23.46 ± 5.97	27.16 ± 6.44	26.23 ± 3.01	25.97 ± 4.22	28.15 ± 2.97	27.27 ± 2.35
rel. liver (%)	2.27 ± 0.35	2.58 ± 0.40	2.41 ± 0.16	2.48 ± 0.37	2.58 ± 0.14	2.53 ± 0.15

Control: no OTA or Se; Se 0.3: 0.3 mg/kg Se; Se 0.5: 0.5 mg/kg Se; OTA: 2 mg/kg OTA; OTA-Se 0.3: 0.3 mg/kg Se and 2 mg/kg OTA; OTA-Se 0.5: 0.5 mg/kg Se and 2 mg/kg OTA.

**Table 2:** Hepatic dataset including phosphatide fatty acid of the 6 experimental groups (means of 6 individual data ± standard deviation).

Group	Control		Se 0.3		Se 0.5		OTA	OTA-Se 0.3			OTA-Se 0.5		OTA	Se	OTA × Se
C14:0	0.08 ± 0.01		0.07 ± 0.01		0.07 ± 0.01		0.08 ± 0.02		0.09 ± 0.01		0.09 ± 0.01				
C16:0	20.05 ± 0.36		19.11 ± 1.33		19.32 ± 0.81		18.80 ± 1.10		19.74 ± 0.94		19.13 ± 0.67				
C18:0	24.50 ± 0.51		24.32 ± 1.52		23.97 ± 1.13		25.51 ± 0.90		24.68 ± 0.78		25.55 ± 0.98				
C20:0	0.06 ± 0.01	a	0.08 ± 0.01	b	0.07 ± 0.01	ab	0.07 ± 0.01	ab	0.08 ± 0.01	ab	0.07 ± 0.01	ab	NS	0.09	0.0001
C22:0	0.06 ± 0.01	ab	0.07 ± 0.01	b	0.07 ± 0.01	b	0.05 ± 0.01	a	0.06 ± 0.01	ab	0.05 ± 0.01	a	0.01	0.037	NS
C23:0	0.02 ± 0.00		0.02 ± 0.01		0.02 ± 0.01		0.02 ± 0.00		0.01 ± 0.00		0.01 ± 0.00				
C16:1n7	1.99 ± 0.60	a	2.99 ± 0.52	b	2.62 ± 0.61	ab	2.04 ± 0.53	ab	2.57 ± 0.69	ab	2.47 ± 0.31	ab	NS	0.04	NS
C18:1n7	1.51 ± 0.26		1.57 ± 0.31		1.62 ± 0.33		1.52 ± 0.15		1.57 ± 0.17		1.68 ± 0.26				
C18:1n9	16.65 ± 2.94	a	20.23 ± 2.40	ab	20.00 ± 2.13	ab	18.97 ± 1.99	ab	21.38 ± 0.75	b	18.89 ± 2.14	ab	NS	NS	NS
C20:1n9	0.32 ± 0.04		0.32 ± 0.05		0.37 ± 0.07		0.38 ± 0.02		0.37 ± 0.03		0.35 ± 0.03				
C22:1n9	0.03 ± 0.01		0.03 ± 0.00		0.03 ± 0.00		0.03 ± 0.00		0.03 ± 0.00		0.03 ± 0.00				
C18:2n6	14.70 ± 0.60		15.33 ± 0.70		14.54 ± 1.02		15.14 ± 0.91		13.99 ± 0.96		14.41 ± 1.31				
C18:3n6	0.24 ± 0.05		0.23 ± 0.09		0.25 ± 0.06		0.17 ± 0.03		0.18 ± 0.03		0.18 ± 0.03				
C20:2n6	0.08 ± 0.05		0.07 ± 0.02		0.06 ± 0.02		0.10 ± 0.04		0.07 ± 0.02		0.06 ± 0.03				
C20:3n6	3.15 ± 0.33		2.97 ± 0.36		3.15 ± 0.35		3.47 ± 0.23		3.06 ± 0.27		3.15 ± 0.24				
C20:4n6	12.77 ± 2.55	b	10.08 ± 1.43	a	11.07 ± 1.30	ab	10.60 ± 1.63	ab	9.59 ± 0.63	a	10.58 ± 0.87	ab	0.016	0.017	NS
C22:4n6	0.93 ± 0.31	b	0.70 ± 0.13	ab	0.75 ± 0.12	ab	0.70 ± 0.09	ab	0.63 ± 0.06	a	0.70 ± 0.09	ab	0.01	0.021	NS
C22:5n6	1.05 ± 0.49	b	0.51 ± 0.07	a	0.60 ± 0.17	a	0.74 ± 0.22	ab	0.59 ± 0.10	a	0.71 ± 0.10	ab	NS	0.01	0.024
C18:3n3	0.10 ± 0.01		0.11 ± 0.01		0.10 ± 0.02		0.10 ± 0.02		0.10 ± 0.02		0.10 ± 0.03				
C20:5n3	0.44 ± 0.03		0.45 ± 0.07		0.51 ± 0.05		0.46 ± 0.04		0.46 ± 0.04		0.44 ± 0.05				
C22:5n3	0.36 ± 0.14	b	0.25 ± 0.04	ab	0.28 ± 0.05	ab	0.31 ± 0.07	ab	0.23 ± 0.02	a	0.27 ± 0.02	ab	NS	NS	NS
C22:6n3	0.90 ± 0.36	b	0.49 ± 0.03	a	0.53 ± 0.10	a	0.75 ± 0.22	ab	0.52 ± 0.10	a	0.65 ± 0.10	ab	NS	0.0001	NS

Control: no OTA or Se; Se 0.3: 0.3 mg/kg Se; Se 0.5: 0.5 mg/kg Se; OTA: 2 mg/kg OTA; OTA-Se 0.3: 0.3 mg/kg Se and 2 mg/kg OTA; OTA-Se 0.5: 0.5 mg/kg Se and 2 mg/kg OTA. Different letters (a, b) in the same row mean significant difference at *p* < 0.05 level. Means with a common letter (ab) do not differ significantly from those groups. NS = not significant.

**Table 3:** Summary of fatty acid classes and lipid quality indices in broiler chicken hepatic total PLs of the 6 experimental groups (means of 6 individual data ± standard deviation).

Group	Control		Se 0.3	Se 0.5		OTA			OTA-Se 0.3	OTA-Se 0.5			OTA	Se	OTA × Se
SFA	44.77 ± 0.40	bc	43.67 ± 1.11	ab	43.53 ± 0.67	a	44.53 ± 0.65	abc	44.65 ± 0.31	abc	44.90 ± 0.61	c	0.041	0.032	0.007
MUFA	20.51 ± 3.72	a	25.14 ± 2.98	b	24.63 ± 2.64	ab	22.94 ± 2.25	ab	25.92 ± 1.35	b	23.42 ± 2.28	ab	NS	0.005	NS
PUFA	34.72 ± 3.67	b	31.18 ± 2.19	ab	31.84 ± 2.45	ab	32.54 ± 2.08	ab	29.42 ± 1.51	a	31.68 ± 1.83	ab	0.05	0.001	NS
n6	32.92 ± 3.22	b	29.89 ± 2.07	ab	30.43 ± 2.33	ab	30.92 ± 1.87	ab	28.11 ± 1.40	a	30.23 ± 1.72	ab	0.044	0.015	NS
n3	1.80 ± 0.48	b	1.30 ± 0.13	a	1.41 ± 0.14	ab	1.61 ± 0.23	ab	1.32 ± 0.12	a	1.45 ± 0.15	ab	NS	0.001	NS
n6/n3	18.86 ± 2.77	a	23.08 ± 1.04	b	21.57 ± 0.95	ab	19.33 ± 1.65	a	21.45 ± 1.14	ab	20.98 ± 1.59	ab	NS	0.0001	NS
UI	130.00 ± 12.45	b	117.96 ± 5.55	ab	121.74 ± 6.30	ab	121.86 ± 6.85	ab	114.48 ± 3.68	a	119.60 ± 3.92	ab	0.026	0.008	NS
ACL	18.03 ± 0.11	b	17.92 ± 0.05	ab	17.95 ± 0.07	ab	17.99 ± 0.08	ab	17.91 ± 0.04	a	17.96 ± 0.03	ab	NS	0.012	NS

Control: no OTA or Se; Se 0.3: 0.3 mg/kg Se; Se 0.5: 0.5 mg/kg Se; OTA: 2 mg/kg OTA; OTA-Se 0.3: 0.3 mg/kg Se and 2 mg/kg OTA; OTA-Se 0.5: 0.5 mg/kg Se and 2 mg/kg OTA. SFA: total Saturated Fatty Acids; MUFA: total Monounsaturated Fatty Acids; n6: total omega-6; n3: total omega-3; n6_n3: ratio of total omega-6 fatty acids to total omega-3 fatty acids; UI: Unsaturation Index; ACL: Average Chain Length. Different letters (a, b, c) in the same row mean significant difference at *p* < 0.05 level. Means with a common letter (ab, bc, abc) do not differ significantly from those groups. NS = not significant.

**Table 4:** Effects of statistical interactions of OTA and Se on the hepatic antioxidant and lipid peroxidation parameters of broilers.

Parameter	OTA	Se	OTA × Se
GSH	0.001	NS	NS
GPx	0.0001	0.006	NS
MDA	0.008	NS	0.0001
CD	0.0001	0.016	NS
CT	0.0001	0.046	NS

GSH: Reduced glutathione, GPx: Glutathione peroxidase, MDA: Malondialdehyde, CD: Conjugated dienes, CT: Conjugated trienes.

**Table 5:** The OTA and Se levels in the experimental feed (mg/kg).

Group	OTA	Se
Control	<0.01	0.07
OTA	2.04 ± 0.13	0.07
Se 0.3 + OTA	1.57 ± 0.19	0.29
Se 0.5 + OTA	2.09 ± 0.23	0.59

## Data Availability

The original contributions presented in this study are included in the article/Supplementary Materials. Further inquiries can be directed to the corresponding authors.

## Supplementary Materials

The following supporting information can be downloaded at: https://www.mdpi.com/article/10.3390/toxins17120568/s1, Table S1: Broiler diet composition and nutrient content (Calculated).
