# Supplementary material for: Dose-Dependent but Non-Interactive Effects of Ochratoxin A and Selenomethionine on Hepatic Lipid Metabolism and Oxidative Stress in Broiler Chickens
Source: Toxins (Basel). 2025 Nov 25;17(12):568. doi: 10.3390/toxins17120568 (PMC12737607; doi:10.3390/toxins17120568)
Supplement: Supplementary file 1 [file toxins-17-00568-s001.zip › toxins-3985825-supplementary.pdf]

Table S1. Broiler diet composition and nutrient content (Calculated)

Nutrient	Control	Se 0.3	Se 0.5	OTA	OTA-Se 0.3	OTA-Se 0.5
Metabolizable energy (MJ/kg)	12.66	12.66	12.66	12.66	12.66	12.66
Dry matter (%)	88.76	88.76	88.76	88.76	88.76	88.76
Crude protein (%)	21.25	21.25	21.25	21.25	21.25	21.25
Crude fiber (%)	4.25	4.25	4.25	4.25	4.25	4.25
Lysine (%)	1.12	1.12	1.12	1.12	1.12	1.12
Methionine (%)	0.38	0.38	0.38	0.38	0.38	0.38
Methionine+cysteine (%)	0.89	0.89	0.89	0.89	0.89	0.89
Calcium (%)	1.10	1.10	1.10	1.10	1.10	1.10
Sodium (%)	0.25	0.25	0.25	0.25	0.25	0.25
Available phosphorus (%)	0.48	0.48	0.48	0.48	0.48	0.48
Selenomethionine (mg/kg)	0.07	0.29	0.59	0.07	0.29	0.59
OTA (mg/kg)	<0.01	<0.01	<0.01	2.04±0.13	1.57±0.19	2.09±0.23
